# Molecular dynamics simulation of human LOX-1 provides an explanation for the lack of OxLDL binding to the Trp150Ala mutant

**DOI:** 10.1186/1472-6807-7-73

**Published:** 2007-11-07

**Authors:** Mattia Falconi, Silvia Biocca, Giuseppe Novelli, Alessandro Desideri

**Affiliations:** 1 Department of Biology and Center of Biostatistics and Bioinformatics, University of Rome "Tor Vergata", Via della Ricerca Scientifica, Rome, Italy, 00133; 2 Department of Neuroscience and Center of Biostatistics and Bioinformatics, University of Rome "Tor Vergata", Via di Tor Vergata, 135, Rome, Italy, 00133; 3 Department of Biopathology and Diagnostic Imaging and Center of Biostatistics and Bioinformatics, University of Rome "Tor Vergata", Azienda Ospedaliera Universitaria, Policlinico Tor Vergata Viale Oxford 81, Rome, Italy, 00133 and Fondazione Livio Patrizi, Rome, Italy

## Abstract

**Background:**

Dimeric lectin-like oxidized low-density lipoprotein receptor-1 LOX-1 is the target receptor for oxidized low density lipoprotein in endothelial cells. *In vivo *assays revealed that in LOX-1 the basic spine arginine residues are important for binding, which is lost upon mutation of Trp150 with alanine. Molecular dynamics simulations of the wild-type LOX-1 and of the Trp150Ala mutant C-type lectin-like domains, have been carried out to gain insight into the severe inactivating effect.

**Results:**

The mutation does not alter the dimer stability, but a different dynamical behaviour differentiates the two proteins. As described by the residues fluctuation, the dynamic cross correlation map and the principal component analysis in the wild-type the two monomers display a symmetrical motion that is not observed in the mutant.

**Conclusion:**

The symmetrical motion of monomers is completely damped by the structural rearrangement caused by the Trp150Ala mutation. An improper dynamical coupling of the monomers and different fluctuations of the basic spine residues are observed, with a consequent altered binding affinity.

## Background

Low density lipoprotein (LDL) is oxidized in vascular endothelial cells to OxLDL, a highly detrimental product that results in endothelial cell injury and is implicated in the development of atherosclerosis. Vascular endothelial cells also internalize and degrade external OxLDL though the lectin-like oxidized low-density lipoprotein receptor-1 (LOX-1) [1-3]. OxLDL causes vascular endothelial cell activation and dysfunction, resulting in pro-inflammatory responses, pro-oxidative conditions, and apoptosis, all of which are pro-atherogenic. LOX-1 has been characterized as the primary receptor for OxLDL on the surface of vascular endothelial cells and is up-regulated in atherosclerotic lesions [2,3]. Upon recognition of OxLDL, LOX-1 is observed to initiate OxLDL internalization and degradation as well as the induction of a variety of pro-atherogenic cellular responses, including reduction of nitric oxide (NO) release [4], secretion of monocyte chemoattractant protein-1 (MCP-1) [5], production of reactive oxygen species [6], expression of matrix metalloproteinase-1 and -3 [7], monocyte adhesion [5], and apoptosis [8].

LOX-1 is a member of the scavenger receptor family, a structurally diverse group of cell surface receptors of the innate immune system that recognize modified lipoproteins. It is a disulfide-linked homodimeric type II transmembrane protein with a short 34-residue cytoplasmic region, a single transmembrane region, and an extracellular region consisting of an 80-residue domain, predicted to be a coiled coil called "neck domain", followed by a 130-residue C-terminal C-type lectin-like domain (CTLD) [2,9].

**Figure 1:**
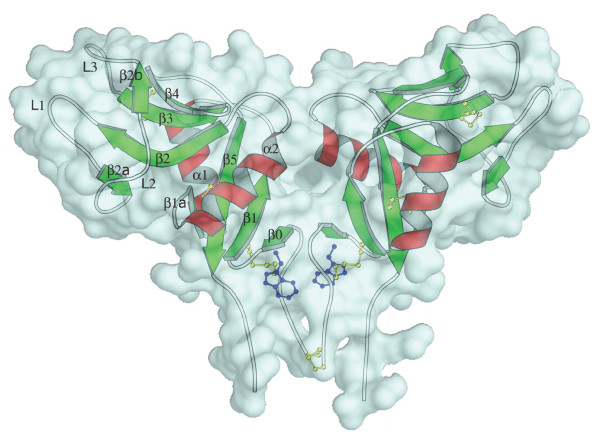
Side view of the LOX-1 CTLD structure. The *α*-helices are shown as red spiral ribbons while *β*-strands are shown as green arrows. The wire regions indicate the random-coil structure and the turns. The side chains of cysteines involved in disulfide bridges and the mutated tryptophan are evidenced by the yellow and blue ball-and-stick representation, respectively. A cyan molecular surface has been superimposed to show the large central cavity and the small cavity below the tryptophans. This picture was produced by using the programs Molscript [36] and Pymol [37].

The crystal structure of the human LOX-1 CTLD has recently been determined [10,11]. Human LOX-1 CTLD forms a heart-shaped homodimer (see Fig. 1), with a tunnel running through the center of the molecule. The LOX-1 monomer has a typical CTLD fold [12] consisting of two antiparallel *β*-sheets, *β*0-*β*1-*β*5-*β*1a and *β*2a-*β*2-*β*3-*β*4-*β*2b, flanked by two *α*-helices, *α*1 and *α*2 (Fig. 1) [10,11]. Three large loops, protruding into the solvent, are included in the second *β*-sheet: L1 from *β*2 to *β*2a, L2 from *β*2a to *β*2b and L3 from *β*2b to *β*3 [10,11]. The fold is further stabilized by three conserved intra-chain disulfide bonds (Cys144-Cys155, Cys172-Cys264 and Cys243-Cys256). A cysteine in position 140, present only in human LOX-1, forms an inter-chain disulfide between the monomers at the N-terminus of the CTLD [10,11]. Deletion analysis has localized OxLDL recognition to the highly conserved (61–83% sequence identity) CTLD of LOX-1 [13].

Several positively charged CTLD LOX-1 residues are known to play a role in the recognition of OxLDL [13-15], and a detailed understanding of this interaction could be of significant medical interest because specific antagonists potentially could mitigate the progression of atherosclerosis. *In vivo *functional assays with LOX-1 mutants revealed that linearly aligned basic residues at the dimer surface, that has been referred as the basic spine (i.e. arginines 208, 229, 231 and 248), are responsible for ligand binding [10]. In fact single elimination of each arginine reduces the binding activity. This effect is even more evident upon mutation of Trp150, a residue located at the dimer interface, into alanine, suggesting that an altered inter-subunit interaction strongly affect the OxLDL binding region [10]. OxLDL has been suggested to have amphipathic *α*-helices on its surface [16], and the basic spine structure of LOX-1 has been proposed to provide an appropriate platform for the interaction with these *α*-helices [10].

In this work we have investigated the not naturally occurring LOX-1 Trp150Ala mutation through molecular dynamics (MD) simulation to study its structural and dynamical properties in comparison to the wild-type protein [10]. Our results show that both the native and mutated proteins have a stable dimeric structure, but they display different overall motion. In the native protein a collective motion generates a symmetrical rotation of each monomer one against the other, while in the mutant this coordinated inter-subunit movement is absent. As a consequence an altered dynamical coupling of the monomers and different fluctuations of the basic spine residues are observed, providing an explanation for the drastic reduction of the OxLDL binding affinity of the mutant protein.

## Results and discussion

### Root Mean Square Deviations and Fluctuations

**Figure 2:**
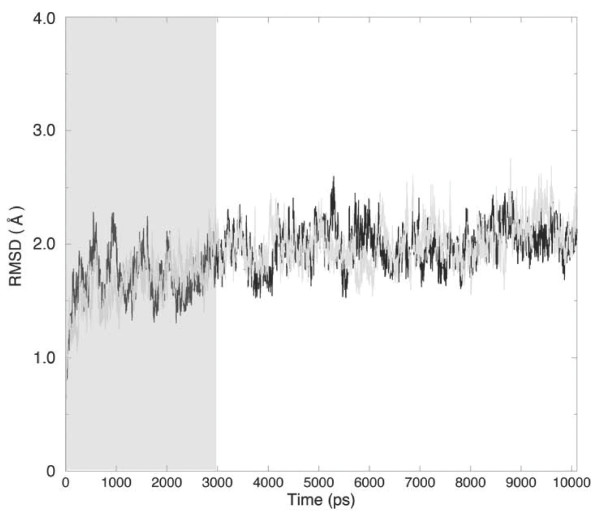
RMSD from starting structures of LOX-1 CTLD wild-type (black line) and Trp150Ala (grey line) proteins. The grey box indicates the trajectory fraction that has not been used in the analysis.

The main chain root mean square deviations (RMSDs) were calculated, for the trajectories of the two proteins, from the starting structures as a function of time (Fig. 2). Although the RMSDs reach a stable value within the first nanosecond all the analyses have been carried out discarding the first three nanoseconds, i.e. over the last seven nanoseconds. This was done to guarantee an investigation over a well thermalized system. Time evolution of the number of residues in *α*-helix, *β*-strand and random coil secondary structures, gyration radius, total solvent accessible surface area (additional file 1) and RMSD (Fig. 1), all confirm the protein stability over the entire trajectory chosen for the analysis.

**Figure 3:**
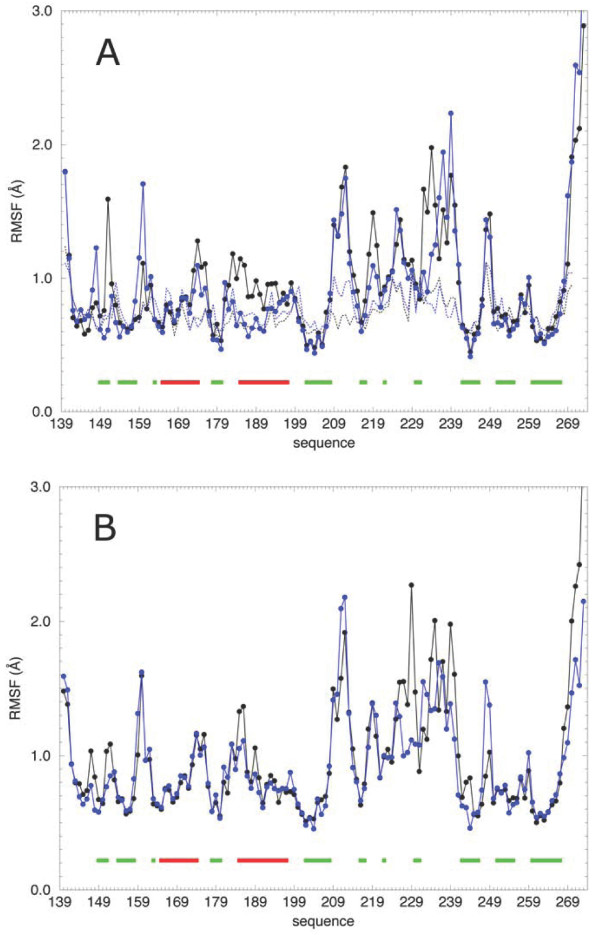
Per residue RMSF of the two subunits of the LOX-1 wild-type **(A) **and Trp150Ala **(B) **proteins. Each residue is indicated by a filled circle. Subunit A is shown by a black line and subunit B by a blue line. The black (subunit A) and blue (subunit B) dotted lines shows the corresponding experimental B-factors as converted to RMSF values from the PDB file 1YPQ (see Eq. 2 in Methods). On the X-axis residues that in the X-ray starting structure are in *α*-helix and *β*-strand are indicated by the red and green bars, respectively.

The main chain root mean square fluctuations (RMSFs) calculated over the trajectories and averaged over each residue for the wild-type and the Trp150Ala (Fig. 3A and 3B), indicate that a large part of residues is characterized by fluctuations not higher than 2.0 Å, apart from the random coil regions of the C-terminal tails which reach values around 3.5 Å. The N-terminal tails are less flexible due to the presence of the inter-subunit disulfide bridge (Cys140.A-Cys140.B) and do not exceed 1.8 Å. A relatively highly fluctuating region in both proteins (values between 1.6 and 2.3 Å) is localized between Arg209 and Gly241, including the loops L1, L2 and L3 and the two small *β*-strands *β*2a and *β*2b.

The two proteins display a similar fluctuation pattern, although important differences are observed for two (i.e. Arg229 and Arg248) of the four residues (i.e. arginines 208, 229, 231 and 248) belonging to the basic spine. In the two subunits of the wild-type the average fluctuation of Arg229 is very similar (1.1 Å in subunit A and 1.0 Å in subunit B, where A and B refer to the order of the monomers given in the PDB file 1YPQ[10]). On the contrary in the mutant this residue is more fluctuating in the first subunit (values are 2.3 Å in subunit A and 1.1 Å in subunit B). A fluctuation difference is observed also for Arg248 that shows a value of 1.4 Å in the two subunits of the wild-type, while it is less fluctuating in the first subunit of the mutant (values are 0.8 Å in subunit A and 1.5 Å in subunit B) (see Table 1).

**Table 1:** RMSF values of the basic spine residues calculated from the simulation of the wild-type and mutant proteins compared to the RMSF values converted from the experimental B-factors.

	**Wild-type RMSF (Å)**		**Trp150Ala RMSF (Å)**		**Converted X-ray B-factors (Å)**	
**Arg.**	Sub. A	Sub. B	Sub. A	Sub. B	Sub. A	Sub. B

**208**	0.8	0.9	0.9	0.9	0.8	0.7
**229**	1.1	1.0	2.3	1.1	1.0	1.1
**231**	0.8	0.9	0.9	1.1	1.0	1.0
**248**	1.4	1.4	0.8	1.5	1.1	1.1

For the native protein the residue RMSF values reproduce well the crystallographic B-factors [10] (Fig. 3A). This is strictly true for the helices and the *β*-strands, while the loops between regular secondary structures segments have fluctuations larger than the corresponding converted B-factors, likely due to the higher degree of hydration of the simulations when compared to the crystal [10]. The B-factor values of basic spine arginines, extracted from the PDB file 1YPQ[10] and converted to RMSF values for comparison (see Methods), are very close to the residue RMSF values detected in the wild-type simulation (see Table 1).

### Secondary structures and cavities

**Figure 4:**
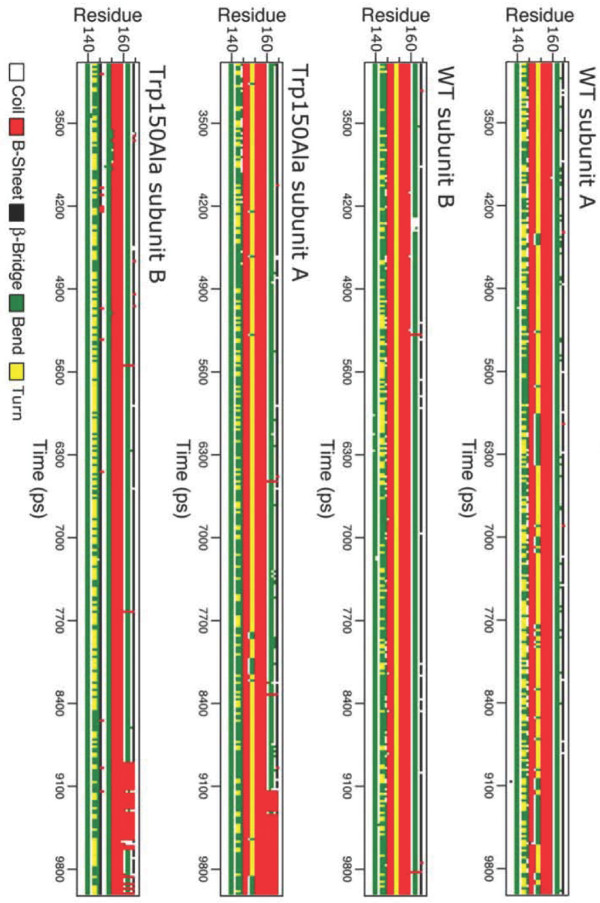
Secondary structure evolution, as a function of time, for the LOX-1 region (140–165) including strands *β*0 (red bar with middle point around 150) and *β*1 (red bar with middle point around 156). Colour code identifying the secondary structure is shown in the figure.

The analysis of the secondary structures, carried out with the program DSSP [17], indicates that the two proteins have comparable secondary structure regions (see also additional file 1). As shown in Fig. 4, a difference is observed only at the level of strand *β*0 (Ile149-His151) involved in the inter-subunit contact. The structure of this *β*-strand is completely lost in one subunit of the mutant.

Two large cavities are present in the LOX-1 CTLD. The first cavity is represented by the "hydrophobic tunnel", which is a 20 Å, mostly non-polar, tunnel localized at the center of the dimer interface [11]. This tunnel is 7–8 Å in diameter except for a constriction that narrows the middle of the tunnel to a diameter of 4 Å [11]. The second cavity, located below the first one and above the inter-chain Cys140.A-Cys140.B disulfide bridge, is smaller and shaped by hydrophobic residues including Pro143, Cys144, Pro145, Trp148, Ile149 and Trp150 [10]. In the mutant protein the amino acid substitution Trp150Ala generates a volume increase of the second cavity (not shown). The volume of the two cavities, monitored along the trajectories of the two proteins by using the program Surfnet [18], is preserved in both simulations.

### Hydrogen bond analysis

The LOX-1 dimer structure shows that Trp150 contributes not only to dimer formation but also to the maintenance of the proper CTLD fold through inter and intra-chain hydrogen bonds [10]. In the wild-type simulation, the maintenance of the short *β*0-*β*1 antiparallel *β*-ribbon is ensured by hydrogen bond network between Trp150.N*ε*1-Gly152.O, Asp147.N-Trp150.O and His151.N-Asn154.O.

In the mutant protein the introduction of an alanine in position 150 disrupts the hydrogen bond between the indole group and Gly152 in both subunits and prevents, in the B subunit, the hydrogen bond between His151 and Asn154, thereby generating the asymmetric unfolding of the *β*0 segment (see Fig. 4). However, new inter-subunit hydrogen bonds arise between Gln146.N*ε*2-Ala150.O and Ala150.N-Trp148.O enforcing the dimeric interactions.

### Cross-correlations and principal component analysis

**Figure 5:**
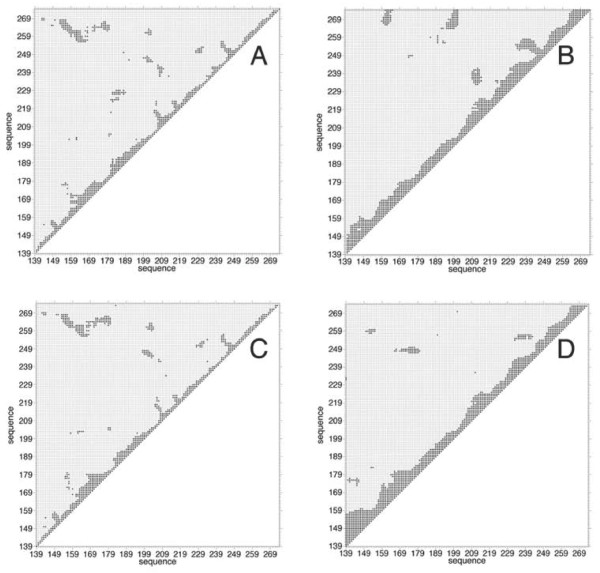
Dynamic cross-correlation maps calculated for the wild-type and the mutant LOX-1 proteins. Panels A and C reports the intra-subunit motion correlations in the wild-type, while panels B and D the intra-subunit motion correlations in the mutant. The black and grey squares represent the C*α *motion correlations with |c_ij_| ≥ 0.5 and |c_ij_| < 0.5, respectively (c_ij _is defined in Eq. 1 of Methods).

Interesting results concerning the relative flexibility and communication of the two proteins can be obtained by looking at the correlated motion between different regions of the protein as described by the dynamic cross correlation (DCC) map calculated on the C*α *atoms [19]. Such plots are reported in Fig. 5, where a black spot represents a correlation between two C_*α *_greater than 0.5 in absolute value. The panels indicate that both the native (panels A and C) and the mutant LOX-1 (panels B and D) have a low degree of correlation. The native protein displays a symmetric behaviour, with the correlation maps being almost identical for the two subunits (panels A and C). In particular, in the wild-type protein the correlation spots present in the two subunits involve the segment including strand *β*1, helix *α*1 and strand *β*1a that is correlated with strand *β*5; strand *β*2 that is correlated with strand *β*3 and *β*5; and strand *β*2b that is correlated with strand *β*4. In contrast the symmetric correlation is lost in the mutant. In this case the maps of the two subunits are different (panels B and D), and an higher degree of correlations is observed between residues adjacent along the sequence (black spots grouped on the diagonal) when compared to the wild-type.

The principal component analysis (PCA), or essential dynamics [20,21], has been also applied to highlight the correlation differences between the native and mutated protein. This analysis is based on the diagonalisation of the covariance matrix built from the atomic fluctuations after the removal of the translational and rotational movement, and permits the identification of the main 3N directions along which the majority of the protein motion is defined. The analysis, carried out on the 268 C*α *atoms of the two proteins, indicates that although the motion is dispersed over 804 eigenvectors, about 80% of the motion depends on the first 30 eigenvectors having the largest eigenvalues (see additional file 2) as generally found in many different systems [22,23].

**Figure 6:**
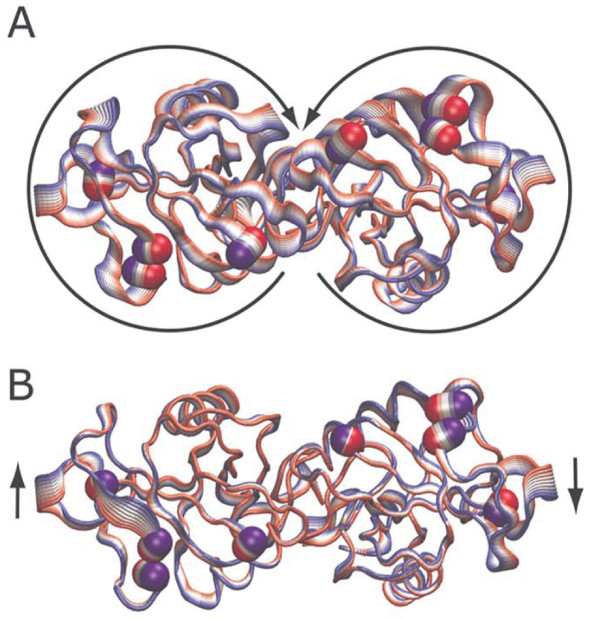
Tube representation of the motion projections along the first eigenvector for the wild-type (A) and Trp150Ala (B) LOX-1 proteins. The direction of the motion is indicated by the arrows and by the flanked tubes, the versus being defined from the red to the blue colour. The C*α *atoms of the basic spine arginines are indicated by the van der Waals spheres using the same colour scheme. This picture was produced using the program VMD [38].

Dynamical differences between the wild-type and mutant proteins can be appreciated looking at the C*α *projections of the MD motions along the first eigenvector, which contain about 20% of the total motion (see additional file 2). The projections of the motion are shown in Fig. 6. The width of the ribbon indicates the amplitude of the backbone motion whilst the direction, evidenced by the arrows, goes from the red to the blue colour. Wild-type LOX-1 (Fig. 6A) shows a symmetrical and uniform rotation of each monomer one against the other, the hinge of this motion being represented by a flexible subunit interface (see also additional file 3).

In mutant LOX-1 (Fig. 6B) the coupling of the inter-subunit motion is cancelled by the mutation that generates a rigid subunit interface and strongly restrains the synchronized motion observed in the wild-type protein. The unique regions having a relative high mobility are now represented by the loops L1, L2 and L3 and the amino and carboxy terminal tails that release the motion gathered by the dimeric structure in the absence of a bendable interface hinge (see also additional file 4). The different motion induces a different behaviour of the residues belonging to the four basic spine arginines of the two subunits, represented by van der Waals spheres in Fig. 6. The arginines, in fact, move in an opposite direction in the two proteins, as indicated by the reverse position of their blue and red colours.

Our analyses indicate that the mutant displays a different dynamical coupling of the monomers, when compared to the native protein, and a different fluctuations of the basic spine arginines, two factors that may prevent the molecular recognition of OxLDL.

## Conclusion

The results obtained from molecular dynamics simulations indicate that both the native and the Trp150Ala mutated protein display a stable dimeric structure that is fully maintained over the entire simulation time. In fact, elimination of the Trp150, located at the inter-subunit interface, mainly induces a dynamical perturbation and only in part a structural rearrangement.

The first important dynamical effect is the occurrence of a different flexibility of two of the four arginine residues (Arg229 and Arg248), which belong to the basic spine (see Fig. 3 and Table 1). These display high flexibility only in one of the two subunits of the Trp150Ala mutant. This asymmetric dynamical behaviour is coupled to the asymmetric destructuration of the *β*0 strand that occurs only in a single subunit of the mutant (see Fig. 4). This is due to the alteration of the hydrogen bond network that, instead, is fully maintained in the native protein. The loss of this short *β*-strand, located at the dimer interface, damps the dimer symmetric motion present in the wild-type as detected through the PCA analysis (Fig. 6A and 6B and additional files 3 and 4). The two monomers in the wild-type undergo a symmetric rotation that pushes the monomers one against the other, using the inter-subunit surface as a flexible hinge (Fig. 6A and additional file 3). On the other hand, in the mutant the inter-subunit surface becomes rigid and the two monomers do not move anymore in a symmetric way (Fig. 6B and additional file 4). This alteration of basic spine dynamical properties disengages the molecular recognition, indicating that the OxLDL needs a regular motion of the monomers for its efficient binding on the receptor surface.

Because the LOX-1 receptor plays a crucial role in atherosclerosis plaque formation, unravelling the molecular mechanism of OxLDL-LOX-1 interaction is of clinical interest. To understand the dynamical aspects of the recognition site could very well be the first step towards the development and therapeutical application of OxLDL antagonists.

## Methods

The human oxidized low density lipoprotein receptor LOX-1 protein coordinates were obtained by X-ray crystallography [10,11] and stored in the Protein Data Bank (PDB) [24]. Five files containing the receptor CTLD are deposited in the PDB; their PDB codes are: 1YPO, 1YPQ, 1YPU[10]1YXJ, 1YXK[11]. The protein coordinates contained in the 1YPQ file, showing the highest resolution (1.4 Å), were chosen to start the simulations. The terminal ends of this structure, due to the absence of the LOX neck domain, are not equally well determined by the X-ray analysis. To avoid excessive motions of the protein tails, that are free to move in the solvent box, the N and C termini of both the monomers have been regularized through molecular modeling. The residues Arg136, Val137, Ala138 and Asn139 have been removed from the N-terminus of monomer B that is four residues longer than the N-terminus of monomer A, and three residues Arg271, Ala272 and Gln273, have been added to the C-terminus of both monomers, following the model coming from the 1YPO LOX-1 structure [10]. In this way the four chain extremities are closer in space and more compact. The dioxane molecule, bound within the largest tunnel chamber, has been removed from the structure, since it is well known that does not induce conformational changes in the protein [10]. The 388 water molecules have been conserved and mixed with those of the simulation boxes built. The homodimer mutant Trp150Ala, and the protein regularization were carried out through the SYBYL 6.0 program [25]. The system topologies have been obtained with the AMBER LeaP module [26], and modelled with the all-atoms AMBER95 force field [27,28]. The proteins have been immersed in rectangular boxes filled with TIP3 water molecules [29] (Table 2), imposing a minimal distance between the solute and the box walls of 10.0 Å. The two systems have been neutralized through the AMBER LeaP module, adding the necessary amount of Cl^- ^ions (Table 2) in electrostatically preferred positions. Two simulations of 10.1 ns of the LOX-1 CTLD have been carried out on the wild-type and the inactive mutant Trp150Ala protein. Optimisation and relaxation of solvent and ions were initially performed by means of three energy minimisations and two molecular dynamics simulations, keeping the solute atoms constrained to their initial position with decreasing force constants of 500 and 25 kcal/(mol Å) (see Table 3). Thereafter the systems were minimised without any constraint and simulated for 160.0 ps at a constant temperature of 300 K using Berendsen's method [30] and at a constant pressure of 1 bar with a 2.0 fs time step (*Δt*). Pressure and temperature coupling constants were 0.4 ps. The atomic positions were saved every 250 steps (0.5 ps) for the analysis. The two systems have been simulated in periodic boundary conditions, using a cut-off radius of 9.0 Å for the non-bonded interactions, and updating the neighbour pair list every 10 steps. The electrostatic interactions were calculated with the Particle Mesh Ewald method [31,32]. The SHAKE algorithm [33] was used to constrain all bond lengths involving hydrogen atoms. The systems were simulated at CASPUR research center of Rome, Italy (Inter Universities Consortium for Supercomputing Applications) on Power 4 IBM parallel computers by using an 8 CPU cluster. The volume of the internal cavities that open between the two subunits along the trajectories of the two simulated proteins was evaluated using the program Surfnet [18], and averaged for a total of 2828 snapshots (1 each 5 saved configurations) extracted from the trajectories. The hydrogen bond analysis was iteratively carried out on the trajectories using an in-house written program executing the HBPLUS v 3.0 program [34]. Dynamic cross correlation map calculation [19] was carried out on the trajectories using an in-house written code. The extent of correlated motions between residues is indicated by the magnitude of the corresponding correlation coefficient between their C*α *atoms. The cross-correlation coefficient for the displacement of each pair of C*α *atoms *i *and *j *is given by:

**Table 2:** Size, box dimensions and number of damped configurations of the two simulated systems.

Simulation system	Wild-type	Trp150Ala
Total atoms	43358	43396
Protein atoms	4180	4152
Amino acids	268	268
Water molecules	13058	13080
Cl^- ^ions	4	2
Box side X (Å)	76	76
Box side Y (Å)	93	93
Box side Z (Å)	61	61
Configurations dumped	20200	20200

**Table 3:** Thermalization scheme of the two simulated systems.

*Time (ps)*	*Thermalization phases*	*Steps number and Δt*	*Position restraint (kcal/mol·Å)*
0	**EM 1**	10000	500
0	**EM 2**	20000	500
12.5	**MD 1**	25000 – 0.5 fs	500
0	**EM 3**	15000	25
25.0	**MD 2**	25000 – 1.0 fs	25
0	EM 4	10000	-
20.0	MD 3	10000 – 2.0 fs	-
40.0	MD 4	20000 – 2.0 fs	-
100.0	MD 5	50000 – 2.0 fs	**-**

The execution time is reported in the left column. EM indicates an Energy Minimization procedure and MD a Molecular Dynamics procedure. For the initial thermalization steps, evidenced in bold, the values of position restraints are shown in the last column.

cij=〈Δri⋅Δrj〉〈Δri2〉〈Δrj2〉
 MathType@MTEF@5@5@+=feaafiart1ev1aaatCvAUfKttLearuWrP9MDH5MBPbIqV92AaeXatLxBI9gBaebbnrfifHhDYfgasaacPC6xNi=xI8qiVKYPFjYdHaVhbbf9v8qqaqFr0xc9vqFj0dXdbba91qpepeI8k8fiI+fsY=rqGqVepae9pg0db9vqaiVgFr0xfr=xfr=xc9adbaqaaeGacaGaaiaabeqaaeqabiWaaaGcbaGaem4yam2aaSbaaSqaaiabdMgaPjabdQgaQbqabaGccqGH9aqpjuaGdaWcaaqaamaaamaabaGaeuiLdqecbeGae8NCai3aaSbaaeaacqWGPbqAaeqaaiabgwSixlabfs5aejab=jhaYnaaBaaabaGaemOAaOgabeaaaiaawMYicaGLQmcaaeaadaGcaaqaamaaamaabaGaeuiLdqKaemOCai3aa0baaeaacqWGPbqAaeaacqaIYaGmaaaacaGLPmIaayPkJaWaaaWaaeaacqqHuoarcqWGYbGCdaqhaaqaaiabdQgaQbqaaiabikdaYaaaaiaawMYicaGLQmcaaeqaaaaaaaa@4CF4@

where Δ*r*_i _is the displacement from the mean position of the *i*^th ^atom and the symbol ⟨⟩ represent the time average over the whole trajectory.

The principal component analysis [20,21], the RMSD and RMSF analyses, gyration radius and total solvent accessible surface area have been calculated using the GROMACS MD package version 3.1.4 [35]. The residue RMSF have been directly compared to the residue temperature factor obtained from X-ray diffraction that is proportional to the B-factor (*B*):

RMSF=〈(Δr)2〉=3B8π2
 MathType@MTEF@5@5@+=feaafiart1ev1aaatCvAUfKttLearuWrP9MDH5MBPbIqV92AaeXatLxBI9gBaebbnrfifHhDYfgasaacPC6xNi=xI8qiVKYPFjYdHaVhbbf9v8qqaqFr0xc9vqFj0dXdbba91qpepeI8k8fiI+fsY=rqGqVepae9pg0db9vqaiVgFr0xfr=xfr=xc9adbaqaaeGacaGaaiaabeqaaeqabiWaaaGcbaGaemOuaiLaemyta0Kaem4uamLaemOrayKaeyypa0ZaaOaaaeaadaaadaqaamaabmaabaGaeuiLdqKaemOCaihacaGLOaGaayzkaaWaaWbaaSqabeaacqaIYaGmaaaakiaawMYicaGLQmcaaSqabaGccqGH9aqpjuaGdaGcaaqaamaalaaabaGaeG4mamJaemOqaieabaGaeGioaGdcciGae8hWda3aaWbaaeqabaGaeGOmaidaaaaaaeqaaaaa@40C0@

Time evolution of the secondary structures have been evaluated by using the DSSP program [17] as implemented in the GROMACS MD package version 3.1.4 [35].

## Authors' contributions

MF performed Molecular Dynamics simulations, analyses, evaluated the results, and drafted the manuscript. AD, SB and GN helped with evaluation of the results produced and in the refining of the manuscript. All authors read and approved the final manuscript.

## Supplementary Material

Additional file 1 Time evolution of structural parameters. Number of residues in *α*-helix (black line), *β*-strand (red line) and random coil secondary structures (blue line) in the wild-type (A) and in the Trp150Ala mutant (B). Gyration radius (C) of wild-type (black line) and Trp150Ala mutant (red line). Total solvent accessible surface area (D) of wild-type (black line) and Trp150Ala mutant (red line). Click here for file

Additional file 2 Cumulative fluctuation as a function of the eigenvector index [20,21]. The wild-type protein is indicated by black filled circles and the Trp150Ala mutant by red filled circles. Only the first 30 eigenvectors are reported. Click here for file

Additional file 3 Movie representing animation of the projections along the first eigenvector for the wild-type LOX-1 protein. The main chain is represented by the blue tube while the C*α *atoms of the basic spine arginines are indicated by the yellow van der Waals spheres. This video was produced using the program VMD [38]. Click here for file

Additional file 4 Movie representing animation of the projections along the first eigenvector for the Trp150Ala LOX-1 protein. The main chain is represented by the red tube while the C*α *atoms of the basic spine arginines are indicated by the yellow van der Waals spheres. This video was produced using the program VMD [38]. Click here for file
