# Supplementary figures and images for: Molecular dynamics simulation of human LOX-1 provides an explanation for the lack of OxLDL binding to the Trp150Ala mutant
Source: BMC Struct Biol. 2007 Nov 7;7:73. doi: 10.1186/1472-6807-7-73 (PMC2194713; doi:10.1186/1472-6807-7-73)

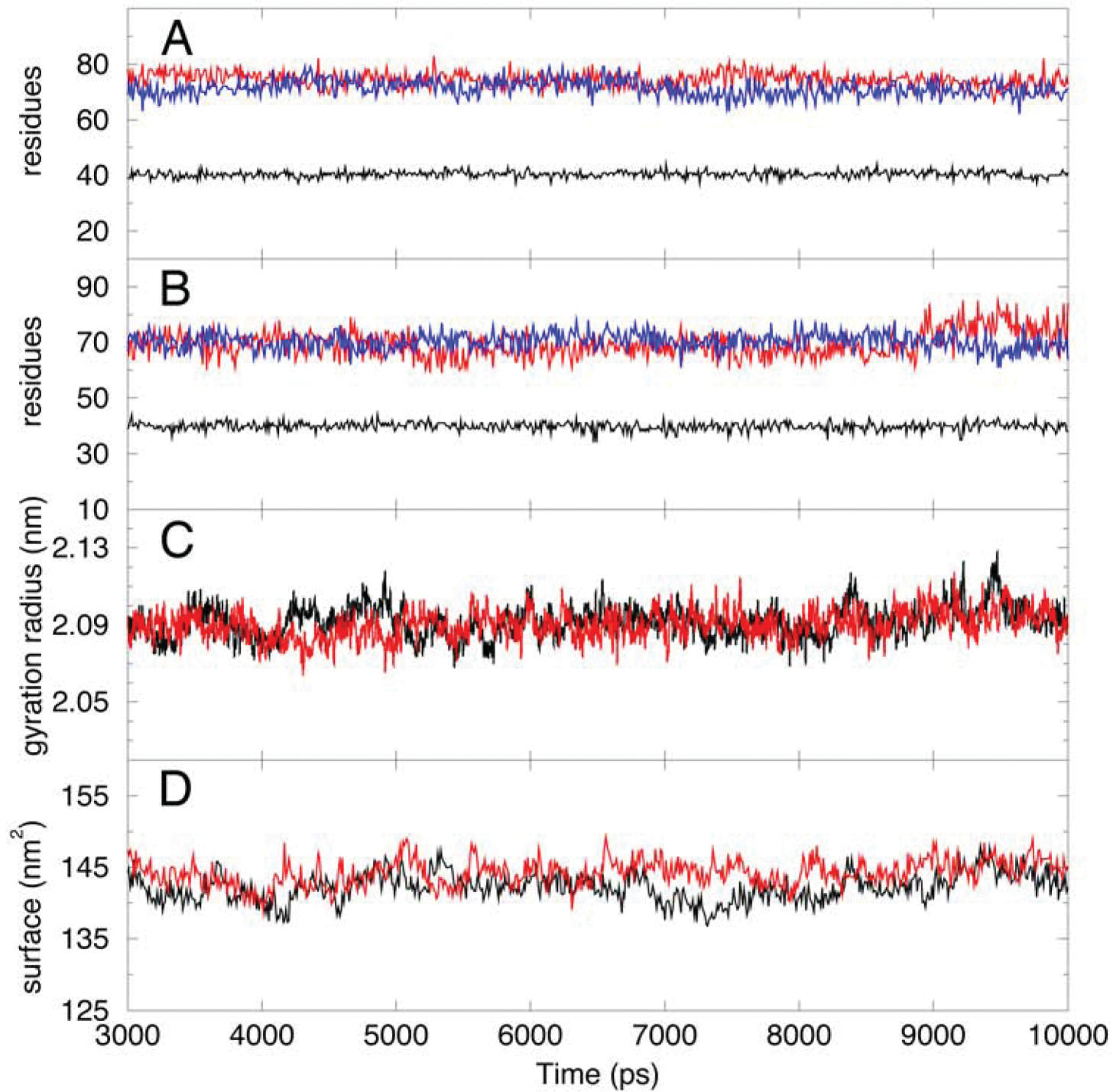

Supplement: Additional file 1 — Time evolution of structural parameters. Number of residues in α-helix (black line), β-strand (red line) and random coil secondary structures (blue line) in the wild-type (A) and in the Trp150Ala mutant (B). Gyration radius (C) of wild-type (black line) and Trp150Ala mutant (red line). Total solvent accessible surface area (D) of wild-type (black line) and Trp150Ala mutant (red line). [file 1472-6807-7-73-S1.pdf]

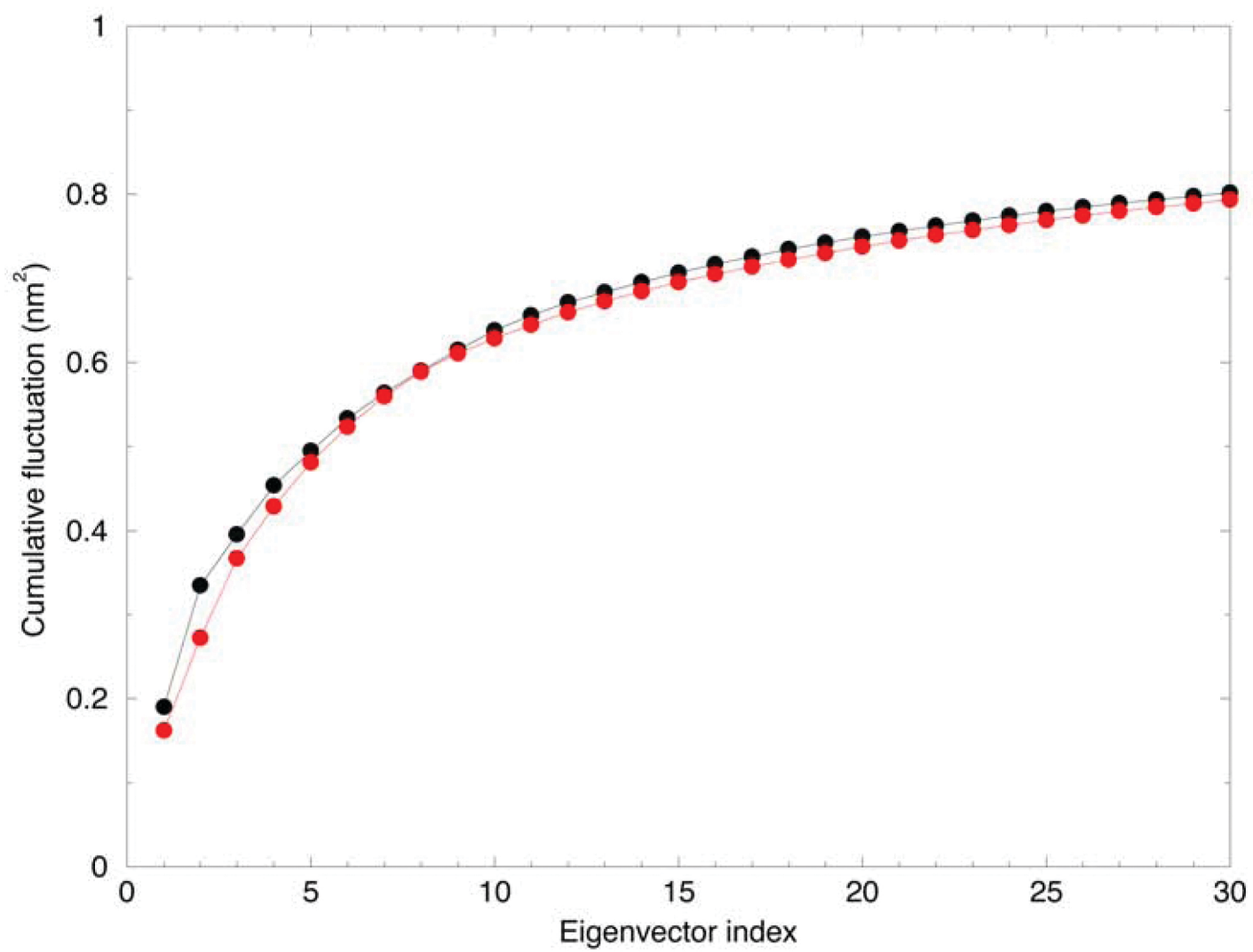

Supplement: Additional file 2 — Cumulative fluctuation as a function of the eigenvector index [20,21]. The wild-type protein is indicated by black filled circles and the Trp150Ala mutant by red filled circles. Only the first 30 eigenvectors are reported. [file 1472-6807-7-73-S2.pdf]
